# Case Report: Evolution of Humoral and Cellular Immunity in Two COVID-19 Breakthrough Infections After BNT162b2 Vaccine

**DOI:** 10.3389/fimmu.2022.790212

**Published:** 2022-02-23

**Authors:** Floriane Gallais, Pierre Gantner, Delphine Planas, Morgane Solis, Timothée Bruel, Florian Pierre, Eric Soulier, Paola Rossolillo, Slim Fourati, Jean Sibilia, Olivier Schwartz, Samira Fafi-Kremer

**Affiliations:** ^1^ CHU de Strasbourg, Laboratoire de Virologie, Strasbourg, France; ^2^ Strasbourg University, Institut National de la Santé et de la Recherche Médicale (INSERM), Unité Mixte de Recherche Scientifique Immuno-Rhumathologie Moléculaire (IRM UMR-S) 1109, Strasbourg, France; ^3^ Virus & Immunity Unit, Department of Virology, Institut Pasteur, Paris, France; ^4^ Centre National de la Recherche Scientifique (CNRS), Unité Mixte de Recherche (UMR) 3569, Paris, France; ^5^ Vaccine Research Institute, Creteil, France; ^6^ Institut de Génétique et de Biologie Moléculaire et Cellulaire, Centre National de la Recherche Scientifique, Unité Mixte de Recherche (UMR) 7104, Institut National de la Santé et de la Recherche Médicale, Université de Strasbourg, Illkirch, France; ^7^ Department of Virology, Hôpital Henri Mondor, Créteil, France; ^8^ Mondor Institute for Biomedical Research (IIMRB), Institut National de la Santé et de la Recherche Médicale (INSERM), Unité Mixte de Recherche (UMR) 955, Créteil, France; ^9^ CHU de Strasbourg, Département de Rhumathologie, Strasbourg, France

**Keywords:** SARS-CoV-2, variant of concern, vaccine, immune evasion, breakthrough infection

## Abstract

**Background:**

SARS-CoV-2 breakthrough infections after complete vaccination are increasing whereas their determinants remain uncharacterized.

**Methods:**

We analyzed two cases of post-vaccination SARS-CoV-2 infections by α and β variants, respectively. For each participant both humoral (binding and neutralizing antibodies) and cellular (activation markers and cytokine expression) immune responses were characterized longitudinally.

**Results:**

The first participant (P1) was infected by an α variant and displayed an extended and short period of viral excretion and symptom. Analysis of cellular and humoral response 72 h post-symptom onset revealed that P1 failed at developing neutralizing antibodies and a potent CD4 memory response (lack of SARS-CoV-2 specific CD4^+^IL-2^+^ cells) and CD8 effector response (CD8^+^IFNγ^+^ cells). The second participant (P2) developed post-vaccination SARS-CoV-2 infection by a β variant, associated with a short period of viral excretion and symptoms. Despite displaying initially high levels and polyfunctional T cell responses, P2 lacked initial β-directed neutralizing antibodies. Both participants developed and/or increased their neutralization activity and cellular responses against all variants, namely, β and δ variants that lasts up to 3 months after breakthrough infection.

**Conclusions:**

An analysis of cellular and humoral response suggests two possible mechanisms of breakthrough infection: a poor immune response to vaccine and viral evasion to neutralizing antibodies.

## Introduction

Since the end of the last year, multiple effective vaccines against the severe acute respiratory syndrome coronavirus 2 (SARS-CoV-2) have been developed in record time to address the COVID-19 pandemic. The BNT162b2 vaccine (Comirnaty, Pfizer-BioNTech) was the first one authorized across the European Union in December and represents today one of the largest supplies of COVID-19 vaccines worldwide. The phase III clinical trial on this mRNA vaccine concluded to an efficacy of 95% of the two-dose regimen against COVID-19, and further observational studies also found efficacy over 90%. According to recent reports, this vaccine keeps its effectiveness against severe COVID-19 linked to escape variants B.1.351 and B.1.617.2 despite a slightly reduced effectiveness against infection. SARS-CoV-2 breakthrough infections after two-dose vaccination are increasing and mainly involve variants of concern (1–3). People of older age or immunocompromised individuals are particularly at risk, especially in the current context of circulation of delta variant (4). However, the mean age of breakthrough cases is now lowering, as younger age groups are now vaccinated.

Here we thoroughly analyzed two cases of mild COVID-19 breakthrough infections diagnosed by reverse transcriptase polymerase-chain-reaction (RT-PCR) on nasopharyngeal swabs at least four weeks after second dose of BNT162b2 vaccine in two women under 50 years of age. Both infecting strains were identified as SARS-CoV-2 variants of concern. Specific humoral and cellular immune responses were characterized early in acute phase of infection to determine in which post-vaccinal immune conditions these infections occurred. These responses were also monitored during the infection course and three months later. Altogether, these observations on multiple virological and immunological aspects would help to better understand physiopathology of breakthrough infections subsequent to complete vaccination.

## Methods

### Patient Disease Histories

Patient 1 (P1) was a 46-year-old woman with obesity (body-mass index = 30.5) despite bypass surgery three years earlier. She received the first dose of BNT162b2 vaccine (Comirnaty^®^) on January 31, 2021 and the second dose on February 21. On March 22 (28 days later), she developed fever, chills, rhinorrhea, sore throat and later muscular pain and cough. She tested positive for SARS-CoV-2 RNA the next day. All symptoms resolved after four weeks except muscle weakness and arthralgia which persisted for three months.

Patient 2 (P2) was a healthy 25-year-old woman. She received the first dose of Comirnaty^®^ on January 8, 2021 and the second dose on January 29. On March 15 (45 days after the second dose), she developed headache, rhinorrhea, loss of smell and night sweats and tested positive for SARS-CoV-2 RNA the same day. A severe fatigue and a slight shortness of breath caused by effort developed in the following days. She completely recovered after two weeks.

### Samples and Data Collection

Nasopharyngeal swabs were sampled at 3, 9, 16, and 85 days after symptom onset (DSO) in P1 and at 0, 10, and 91 DSO in P2. Blood samples were collected at 3–4, 9–10, 16–24, and 85–91 DSO in P1 and P2, respectively. Peripheral blood mononuclear cells (PBMCs) were isolated from the first and the last blood sample collected in each patient and cryo-preserved at −150°C until use. The study was approved by the Ethics Committee of the University Hospital of Strasbourg (N°CE–2020–51). Written informed consent was provided by both participants.

### SARS-CoV-2 RT-PCR Assay

All nasopharyngeal swabs, except the sample collected at diagnosis in P2, were analyzed in our laboratory with SARS-CoV-2 specific primers and probes targeting two regions on the viral RNA-dependent RNA polymerase (RdRp) gene, namely IP2 and IP4 (Institut Pasteur, Paris, France; WHO technical guidance). Cycle thresholds (Ct) values obtained with IP2 target were considered for analyses.

### SARS-CoV-2 Genome Sequencing

Full-length SARS-CoV-2 genome sequencing were performed for both patients on a NextSeq 500 device (Illumina) as previously described (5) (see **Supplementary Methods**). Lineages and clades were interpreted using Pangolin and NextClade, before being submitted to the GISAID database.

### Serological Assays

IgG directed against the SARS-CoV-2 nucleocapsid (N) and the Receptor Binding Domain (RBD) of the spike (S) protein were quantified using the chemiluminescence microparticle immunoassays (CMIA) Abbott Architect SARS-CoV-2 IgG and SARS-CoV-2 IgG Quant II (Abbott), respectively. Anti-S IgG and IgA were measured by S-Flow assay based on the recognition of SARS-CoV-2 S protein expressed on the surface of human embryonic kidney (HEK) 293T cells, as described previously (6) (see **Supplementary Methods**).

### Neutralization Assays

Neutralizing antibody titers were measured for each serum using a viral pseudoparticle-based assay. Pseudoparticles (provided by Rossolillo Laboratory, IGBMC) harboring the S protein of D614G, alpha or beta variants on their surface and encoding for a luciferase reporter gene were preincubated for 1 h at +37°C with serial serum dilutions. The mix were added to 293T-ACE2 target cells (provided by O Schwartz Laboratory, Institut Pasteur) plated in 96-well plates with 30,000 cells per well. The luciferase signal was measured after 72 h incubation at 37°C. Analyses were performed in triplicates and the neutralization titer was defined as the geometric mean log_10_ of the sample dilutions that yielded 50% inhibition of pseudoparticle infectivity (log_10_ IC_50_). Sera were considered neutralizing if the 1:40 dilution (1.60 log_10_) did mediate at least a 50% luminometric signal reduction relative to the control condition without serum.

A live-virus neutralizing assay using the S-Fuse reporter cells was performed using D614G, alpha, beta, and delta variants, as previously described (7). Briefly, S-Fuse U2OS cells expressing ACE2 and either GFP1–10 or GFP11 were infected with SARS-CoV-2 incubated with serial serum dilutions. Infection with SARS-CoV-2 was measured by numbering GFP-producing syncytia formed upon productive infection after 18 h. Neutralization of infectious D614G, B.1.1.7, B.1.351, and B.1.617.2 variants was assessed for each serum. Neutralizing activity of each serum was expressed as the log_10_ of half-maximal inhibitory concentration (IC_50_), with the first serum dilution tested of 1:30 (1.48 log_10_) set as positivity threshold.

### ELISpot Assay

SARS-CoV-2 specific T cell immune response was investigated by an interferon gamma (IFN-γ) enzyme linked immunospot (ELISpot) assay on peripheral blood mononuclear cells (PBMCs). Cells were seeded at 250,000 PBMCs/well and stimulated for 20 ± 4 h in duplicates with overlapping 15-mer peptide pools spanning the N-terminal and C-terminal fractions of SARS-CoV-2 S proteins (S1 and S2, respectively) of the wild-type (WT) SARS-CoV-2 strain and the alpha (α) and beta (β) variants (PepMix™, JPT Peptide Technologies). Cells with AIM-V culture medium (Gibco Life Technologies) were used as negative controls and with phytohemagglutinin-L (PHA, Sigma-Aldrich) as positive controls. PBMCs were cultured overnight (20 ± 4 h) at 37°C before enzymatic revelation of IFN-ɤ capture. Spots were counted using an ELISPOT reader (AID iSpot). Results were expressed as the mean number of spot forming units (SFU)/10^6^ PBMCs after subtraction of the background value. The threshold defining a significant T cell reactivity for one antigen was set at exceeding three standard deviations of the negative control background.

### AIM and ICS Assays

Antigen specific and cytokine-expressing cells were characterized by activation-induced marker (AIM) and intra-cellular staining (ICS) assays (see **Supplementary Methods**). Thawed PBMCs were put in culture at a concentration of 10 million cells/ml. After a rest of 1 h at 37°C, a CD40 blocking antibody was added. After 15 min at 37°C, cells were stimulated with 0.5 μg/ml PHA or 0.44 μg/ml of overlapping peptide pools spanning S1 and S2 of WT, alpha and beta SARS-CoV-2 strains (PepMix™, JPT Peptide Technologies) for 9 h at 37°C allowing for upregulation of CD69/CD40L/OX40 on antigen-specific cells (AIM^+^). Cells were further incubated for 12 additional hours at 37°C in the presence of Brefeldin A (Sigma) to allow for subsequent intracellular cytokines accumulation. An unstimulated condition served as a negative control.

Cells were then collected, stained with the Aqua Live/Dead staining kit (ThermoFisher Scientific), for 20 min at 4°C and then with antibodies against extracellular molecules. After fixation with paraformaldehyde and permeabilization with the PermWash buffer (BD), cells were stained with antibodies targeting intracellular cytokines for an additional 45 min at room temperature. Cells were then washed and resuspended in PBS for subsequent analysis. After acquisition (Navios, Beckman-Coulter), analysis was performed using FlowJo software version 10.7.1.

## Results

**Figure 1:**
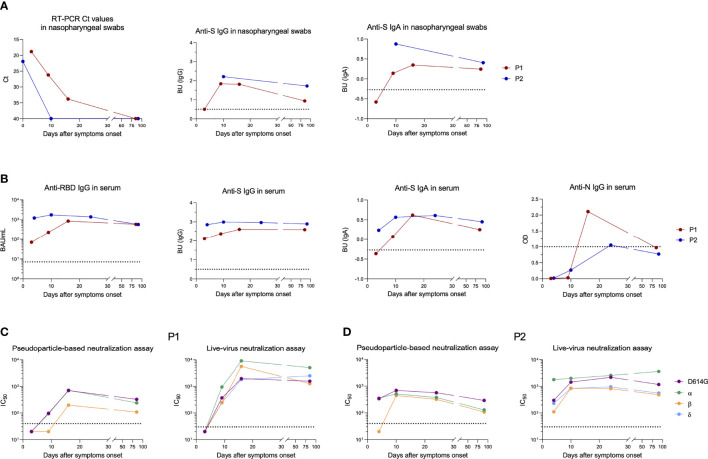
Longitudinal follow-up of anti-SARS-CoV-2 humoral response after breakthrough infection in patient 1 (P1) and patient 2 (P2). **(A)** Viral shedding and antibodies detected against the SARS-CoV-2 spike (S) protein in nasopharyngeal swabs from P1 (red dots) and P2 (blue dots). Ct values are depicted on the first graph (left) and anti-S IgA and IgG levels on the two other graphs. to the positivity threshold. **(B)** Antibodies directed against SARS-CoV-2 receptor binding domain (RBD), S or nucleocapsid (N) proteins measured in serum from P1 (red dots) and P2 (blue dots). **(C, D)** Half-maximal inhibitory concentration (IC_50_) measured in P1 **(C)** and P2 **(D)** by pseudoparticle-based (left) and live-virus neutralization (right) assays against several SARS-CoV-2 variants. Sera displaying less than 50% luminometric signal reduction relative to the control condition with the first serum dilution tested (1:40 and 1:30 with pseudoparticle-based and live-virus neutralization assays, respectively) were considered negative and depicted on graphs with an IC_50_ set arbitrarily at 20. The dotted horizontal black lines correspond to positivity thresholds. BAU, Binding Antibody Units; BU, binding units; Ct, Cycle threshold.

The nasopharyngeal swab from P1 showed a high viral load of alpha variant (cycle threshold (Ct) = 18.8) at 3 DSO, that lasted until 16 DSO (Ct: 33.8) (**Figure 1A**). Mucosal anti-S IgA and IgG were negative at 3 DSO, appeared at 9 DSO and persisted until day 85 (**Figure 1A**). Serum sample collected at 3 DSO was positive for anti-RBD (70 BAU/ml) and anti-S IgG (**Figure 1B**). This serum failed to neutralize any of the viral strains (**Figure 1C**). At 16 DSO, Anti-RBD IgG increased by 1.08 log_10_ (846 BAU/ml), neutralization titers reached over 2 log_10_ IC_50_ against the 4 viral strains (D614G, alpha, beta, and delta) and persisted until 85 DSO. Anti-S IgA and anti-N IgG appeared at 9 and 16 DSO, respectively (**Figure 1B**). Anti-S IgA persisted during follow-up while anti-N IgG became negative after three months.

At the time of diagnosis, P2 displayed a high viral load of beta variant (Ct = 21.9) (**Figure 1A**). Because of the presence of lysis buffer in transport medium of the nasopharyngeal swab collected at the day of diagnosis, we were not able to analyze anti-S IgG and IgA. On day 10, anti-S IgG and IgA were positive in the nasopharyngeal swab and RNA became undetectable (**Figure 1A**). Anti-RBD IgG (1,220 BAU/ml) and also anti-S IgG and IgA were positive in serum at 4 DSO, whereas no anti-N IgG were detected (**Figure 1B**). At this time, neutralization was negative against beta variant with pseudoparticle-based assay and weak (2.04 log_10_ IC_50_) with live-virus neutralization assay (**Figure 1D**). Serum anti-RBD IgG slightly increased by day 10 (1,739 BAU/ml) and neutralizing antibody titer reached at least 2.6 log_10_ IC_50_ against beta variant and persisted up to 3 months against all tested variants (**Figure 1D**).

**Figure 2:**
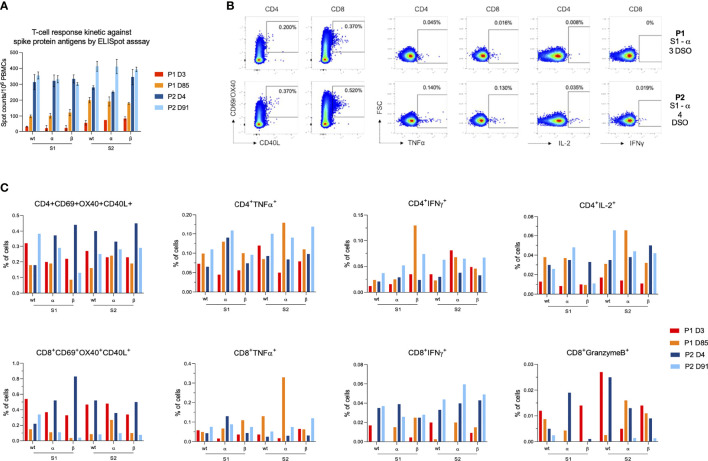
Characterization of anti-SARS-CoV-2 cellular response after breakthrough infection in patient 1 (P1) and patient 2 (P2). **(A)** T-cell reactivity measured by IFN-γ ELISPOT against the N-terminal (S1) and C-terminal (S2) parts of the SARS-CoV-2 spike protein from wild-type strain and α and β variants. Bar charts and error bars represent mean positive values and standard deviations of spot counts per million of peripheral blood mononuclear cells (PBMCs). **(B)** Dot plots showing examples of AIM^+^ and cytokine^+^ cells after stimulation with S1 α peptide pools on the early infection time points for P1 (first row) and P2 (second row). From left to right, dot plots depict, CD4^+^AIM^+^, CD8^+^AIM^+^, CD4^+^TNFα^+^, CD8^+^TNFα^+^, CD4^+^IL-2^+^ and CD8^+^IFNγ^+^ cells. **(C)** Bar charts representing AIM^+^ and cytokine^+^ cells frequencies for each T cell subset (either CD4 or CD8). Results are displayed for each variant (wild type, α and β) and each SARS-CoV-2 part (S1 and S2). The color code is as follows: P1 at 3 days (red) and 85 days (orange) and from P2 at 4 days (dark blue) and 91 days (light blue) after symptom onset.

In contrast to humoral response, SARS-CoV-2 specific T cell response was not different between viral variants. Early after infection, the frequency of IFN-γ-producing T cells directed against the S protein was higher in P2 (mean S1 ± standard deviation (SD) between all variants: 323 ± 31 SFU/10^6^ PBMCs, S2: 293 ± 49 SFU/10^6^ PBMCs) than that observed in P1 (S1: 24 ± 11 SFU/10^6^ PBMCs, S2: 70 ± 16 SFU/10^6^ PBMCs) (**Figures 2A**, **S1**). Low frequency of CD8^+^IFNγ^+^ cells were also observed by ICS assay in P1 while CD4^+^IFNγ^+^ T cells and also those expressing TNFα were detected at similar frequencies between participants (**Figures 2B, C**, **S2**). Interestingly, IL-2-expressing CD4^+^ T cells were also detected at lower frequencies in P1 compared to P2. P1 tended to display lower rates of antigen-specific CD4^+^ (CD4^+^AIM^+^, CD69^+^OX40^+^CD40L^+^) cells than P2 (ranging between 0.20–0.32 and 0.18–0.45%, respectively), but both displayed similar rates of CD8^+^AIM^+^ cells (ranging between 0.22 and 0.83%). Three months later, CD4^+^AIM^+^ frequencies remained slightly lower in P1 while CD8^+^AIM^+^ frequencies decreased to reach the same range (0.03–0.11%) in both participants. Conversely, the frequency of S-specific IFN-γ-producing T cells (CD4^+^ and CD8^+^ cells) measured by ELISpot increased after three months for P1 both against S1 (107 ± 16 SFU/10^6^ PBMCs) and S2 (191 ± 17 SFU/10^6^ PBMCs) (**Figures 2A**, **S1**). For P2, IFN-γ responses increased against S2 (407 ± 27 SFU/10^6^ PBMCs) but remained stable against S1 (330 ± 28 SFU/10^6^ PBMCs). Both participants displayed polyfunctional cytokine responses (TNFα^+^, IFNγ^+^, IL-2^+^) in both subsets at higher levels than early in infection (**Figures 2B, C**, **S2**).

## Discussion

In this study, we report two cases of mild breakthrough infection by alpha and beta variants, respectively. Both patients displayed similar viral loads at symptom onset, however, viral shedding and clinical symptoms evolve differently. The analysis of cellular and humoral response in both cases suggests two possible mechanisms of breakthrough infection, a poor immune response to vaccine (P1) and viral evasion to neutralizing antibodies (P2).

Early after symptom onset, analysis of P1 samples revealed the absence of mucosal anti-S antibodies and a low level of circulating anti-S antibodies that failed to neutralize all tested variants. In addition, P1 displayed low levels of CD8^+^ IFNγ^+^ cells and also CD4^+^IL2^+^ cells, suggesting a low level of circulating effector CD8^+^ T cells and of CD4^+^ central memory T cells, respectively. This poor response may explain the relatively long period of viral shedding and persistence of COVID-19 symptoms up to 3 months DSO. Underlying obesity of this immunocompetent patient may explain the poor immune response after two doses of vaccine although recent studies dispute the role of obesity in the immune response to vaccination (8, 9). Breakthrough infection of P2 appears to be due to escape of the beta variant from neutralizing antibodies irrespective of SARS-CoV-2 specific T cells levels, consistent with previous studies (10, 11). Interestingly, both patients developed neutralization activity against all variants including beta and delta variants that lasts up to 3 months after breakthrough infection.

The limitation of this study relies on unavailability of blood sample before breakthrough infection in both patients. Instead, post-vaccinal SARS-CoV-2 immune responses were assessed in the three to four days following the first symptoms onset and were therefore possibly already boosted by anamnestic responses. However, we revealed a lack of neutralizing antibodies against variants involved in infection in both patients, which was undoubtedly already the case before infection. Moreover, no anti-N antibodies were detected consequently to infection at this time and only low antibody levels were found in P1, suggesting that these immune responses measured early after infection were a good reflection of the previous vaccine response. Finally, we did not explore *de novo* T cell responses induced by infection (particularly against nucleocapsid and membrane proteins) that may also have shortened the time of viral clearance and tempered disease severity, along with anti-S T cell recall responses (12–14).

## Data Availability Statement

The datasets presented in this study can be found in online repositories. The names of the repository/repositories and accession number(s) can be found below: https://www.ncbi.nlm.nih.gov/, accession ID: OM714898 and OM702711.

## Ethics Statement

The studies involving human participants were reviewed and approved by the Ethics Committee of the University Hospital of Strasbourg (N°CE–2020–51). The patients/participants provided their written informed consent to participate in this study.

## Author Contributions

FG, PG, OS and SFK contributed to conception and design of the study. FG, PG, DP, TB, FP and ES performed the experiments. PR contributed to design the experiments. MS, SF, and JS helped at data collection. FG and PG organized the database. FG and PG performed the statistical analysis. FG wrote the first draft of the manuscript. PG and SFK wrote sections of the manuscript. All authors listed have made a substantial, direct, and intellectual contribution to the work and approved it for publication.

## Funding

This work was supported by the Strasbourg University Hospital (SeroCoV-HUS; PRI 7782), ANR-18-CE17-0028, ANR-11-LABX-0070_TRANSPLANTEX, Strasbourg University and the Institut National de la Santé et de la Recherche Médicale (UMR_S 1109). Work in OS lab is funded by the Institut Pasteur, Urgence COVID-19 Fundraising Campaign of Institut Pasteur, Fondation pour la Recherche Médicale (FRM), ANRS, the Vaccine Research Institute (ANR-10-LABX-77), Labex IBEID (ANR-10-LABX-62-IBEID), ANR/FRM Flash Covid PROTEO-SARS-CoV-2 and IDISCOVR. DP is supported by the Vaccine Research Institute.

## Conflict of Interest

The authors declare that the research was conducted in the absence of any commercial or financial relationships that could be construed as a potential conflict of interest.

## Publisher’s Note

All claims expressed in this article are solely those of the authors and do not necessarily represent those of their affiliated organizations, or those of the publisher, the editors and the reviewers. Any product that may be evaluated in this article, or claim that may be made by its manufacturer, is not guaranteed or endorsed by the publisher.
