# Supplementary material for: Case Report: Evolution of Humoral and Cellular Immunity in Two COVID-19 Breakthrough Infections After BNT162b2 Vaccine
Source: Front Immunol. 2022 Feb 23;13:790212. doi: 10.3389/fimmu.2022.790212 (PMC8905643; doi:10.3389/fimmu.2022.790212)
Supplement: Supplementary file 1 [file DataSheet_1.docx]

 
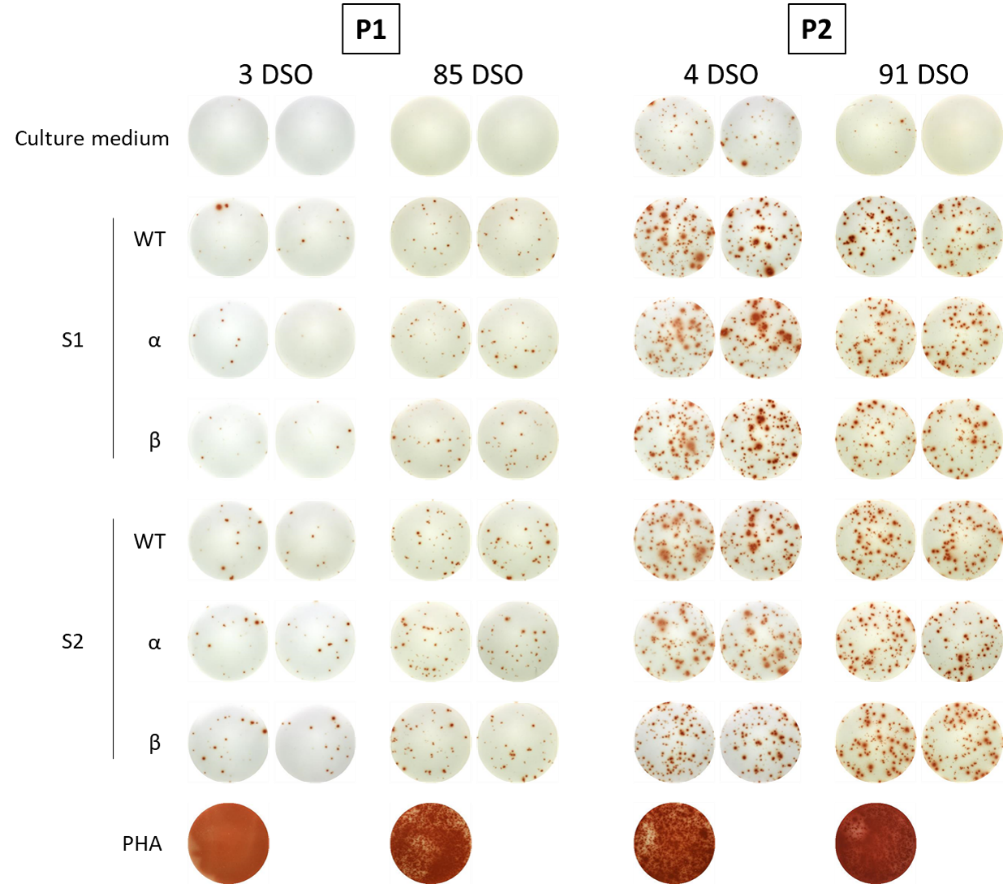


**Supplementary Figure S1. Images obtained by IFN-γ ELISPOT conducted on peripheral blood cells isolated from patient 1 (P1) and from patient 2 (P2) after breakthrough infection.** Cells were collected in P1 at 3 and 85 days after symptoms onset (DSO) (left) and in P2 at 4 and 91 DSO (right). T cell specific responses were evaluated using peptide pools spanning the SARS-CoV-2 spike protein (N-terminal region = S1, C-terminal region = S2) of the reference strain (wild-type, wt) and of α and β variants. All experiments were performed in duplicates, with at least two wells of negative controls (cells with culture medium only) and one well of positive control (phytohemagglutinin) for each sample. P2 was highly reactive to all peptide pools irrespective of the variants at 4 DSO whereas P1 was only poorly reactive. T-cell response was increased in P1 at 85 DSO but remained lower than those measured in P2 at 91 DSO.

**Supplementary Figure S2. Gating strategy.** Gating strategy used for AIM/ICS analysis, with examples of dot plots showing cytokine+ and AIM+ cells after stimulation (row “+”) and unstimulated cells as negative controls (row “-“).

**Supplementary methods**

**Full-length SARS-CoV-2 genome sequencing**

The full-length SARS-CoV-2 genomes were sequenced by means of next-generation sequencing. Briefly, viral RNA was extracted from nasopharyngeal swabs in viral transport medium using NucliSENS® easyMAG kit on EMAG device (bioMérieux, Marcy-l’Étoile, France). Sequencing was performed with the Illumina COVIDSeq Test (Illumina, San Diego, California), that uses 98-target multiplex amplifications along the full SARS-CoV-2 genome. The libraries were sequenced with NextSeq 500/550 High Output Kit v2.5 (75 Cycles) on a NextSeq 500 device (Illumina). The sequences were demultiplexed and assembled as full-length genomes by means of the DRAGEN COVIDSeq Test Pipeline on a local DRAGEN server (Illumina). Lineages and clades were interpreted using Pangolin and NextClade, before being submitted to the GISAID database (<https://www.gisaid.org>).

***Serology S-flow assay***

Anti-S IgG and IgA were measured by S-Flow assay based on the recognition of SARS-CoV-2 S protein expressed on the surface of human embryonic kidney (HEK) 293T cells. Briefly, 293T cells (ATCC® CRL-3216™) were transfected with the indicated S-protein expression plasmids or a control plasmid using Lipofectamine 2000 (Life Technologies). One day after, transfected cells were detached using PBS-EDTA and transferred into U-bottom 96-well plates (50,000 cells per well). Cells were incubated at 4 °C for 30 min with sera (1:300 dilution) or nasal swabs (1:50 dilution) in PBS containing 0.5% BSA and 2 mM EDTA, washed with PBS and stained using anti-IgG AF647 (1:600 dilution; Thermo Fisher). Cells were washed with PBS and fixed for 10 min using 4% paraformaldehyde. Data were acquired on an Attune Nxt instrument (Life Technologies). Stainings were also performed on control (293T-empty) cells. The specific binding was calculated as follow: 100 x (% binding 293T Spike - % binding 293T 227 Empty)/ (100 - % binding 293T Empty). The positivity of a sample was defined as a specific binding above 40% for IgG and 20% for IgA. The calculation of Binding units (BU) enabled to standardize the results by establishing a dose-response curve with a reference anti-Spike human monoclonal antibody.

**AIM and ICS assays**

Antigen specific and cytokine-expressing cells were characterized by activation-induced marker (AIM) and intra-cellular staining (ICS) assays. PBMCs were thawed, washed and put in culture at a concentration of 10 million cells/mL in RPMI 1640 medium (Gibco Life Technologies, Cat# 11,875-093) supplemented with 0.5% penicillin/streptomycin (Gibco, Cat# 15070063) and 10% human serum (Sigma, Cat# H5522-100mL). After a rest of 1 h at 37 °C, a CD40 blocking antibody (Miltenyi-MACS, Cat# 130-094-133) was added to the culture to prevent the interaction of CD40L with CD40 and its subsequent downregulation. After 15 min incubation at 37°C, cells were stimulated with 0.5 μg/mL PHA or 0.44 μg/ml of overlapping peptide pools spanning S1 and S2 of WT, α and β SARS-CoV-2 strains (PepMix™, JPT Peptide Technologies, Cat# PM-WCPV-S, PM-SARS2-SMUT01-1, PM-SARS2-SMUT02-1, respectively) for 9 hours at 37°C allowing for upregulation of CD69/CD40L/OX40 on antigen-specific cells (AIM+). Cells were further incubated for 12 additional hours at 37°C in the presence of Brefeldin A (BFA, Sigma, Cat# 7651). An unstimulated condition served as a negative control.

After resting or stimulation, cells were collected, resuspended in PBS and stained with the Aqua Live/Dead staining kit (ThermoFisher Scientific, Cat#L34957) for 20min at 4°C. Cells were then stained with antibodies against extracellular molecules in PBS + 4% human serum for 20min at 4°C. After a 15min fixation with PBS + 4% paraformaldehyde (Sigma, Cat# 252549), a permeabilization step was performed with the PermWash buffer for 30min (BD, Cat# 554723) following the manufacturer’s instructions. Cells were then stained with antibodies targeting intracellular cytokinesfor an additional 45min at room temperature in the PermWash Buffer. Cells were then washed and resuspended in PBS for subsequent analysis. After acquisition (Navios, Beckman-Coulter), analysis was performed using FlowJo software version 10.7.1 (Tree Star, Ashland, OR).

Antibodies used: CD4-APC-H7 (Clone: RPA-T4, Cat#560168, Dilution 1/100), CD3-A700 (Clone: UCHT1, Cat#557943, Dilution 1/200), CD69-BV786 (Clone: FN50, Cat#563834, Dilution 1/50), OX40-BV605 (Clone: ACT35, Cat#743284, Dilution 1/50), IFNγ-PE-Cy7 (Clone: B27, Cat#557643, Dilution 1/100), TNFα-APC (Clone: MAb11, Cat#562084, Dilution 1/100), GranzymeB-PE (Clone: GB11, Cat#561142, Dilution 1/33) and IL-2-PerCP-Cy5.5 (Clone: MQ1-17H2, Cat#560708, Dilution 1/33) and CD40L-BV421 (Clone: TRAP1, Cat#563886, Dilution 1/25) purchased from BD Bioscience, and CD8-FITC (Clone: BW135/80, Cat#130-113-719, Dilution 1/200) from Miltenyi/MACS.
